# Supplementary material for: Patient and health system related factors affecting diagnostic and treatment delays in tuberculosis: A cross-sectional study in Mysuru, South India
Source: PLoS One. 2026 Feb 12;21(2):e0342998. doi: 10.1371/journal.pone.0342998 (PMC12900347; doi:10.1371/journal.pone.0342998)
Supplement: S1 File — (PDF) [file pone.0342998.s001.pdf]

Factors Predicting Diagnostic Delays in Tuberculosis: A cross-sectional analysis of Patient Demographics, Comorbidities, and Healthcare Pathways

Research Questionnaire (English)

Demographic Information

1. Personal Details:	
**Participant ID:**	
**Nikshay ID:**	
**Age:**	_____ year<br><br>DoB: ______________________ (yy/mm/dd)
**Gender:**	☐ Male ☐ Female ☐ Other
**BMI:**	Height: ______ cm<br>Weight: ______ kg
**Religion:**	☐ Hindu<br>☐ Muslim<br>☐ Christian<br>☐ Other — ___________
**Marital Status:**	☐ Single ☐ Married ☐ Widowed ☐ Divorced
**Type of Family**	☐ Nuclear ☐ Joint / Extended
**Family size (Members)**	☐ 1 to 4 ☐ 5 to 7 ☐ >8
**Place of Residence/Location:**	☐ Urban ☐ Rural
**2. Socio-economic status:**	
**Education:**	☐ Illiterate<br>☐ Primary (1 to 5th standard)<br>☐ Middle school (6th to 8th standard)<br>☐ Secondary (9th to 10th standard)<br>☐ Senior secondary (11th to 12th / PUC)<br>☐ Graduate
**Occupation/Employment:**	☐ Unemployed<br>☐ Agriculture<br>☐ Salaried<br>☐ Business<br>☐ Daily wage / Casual<br>☐ Housewife<br>☐ Dependent<br>☐ Student<br>☐ Other ___________
**Economic condition:**	☐ Below Poverty Line (BPL)<br>☐ Above Poverty Line (APL)<br>☐ Not available
**Health Insurance Enrolment Status**	☐ Yes ☐ No
**3. Smoking History:**	

**Smoker:**	☐ Current smoker<br>☐ Quit smoking<br>☐ Non-smoker (never)
**4. Alcohol Consumption:**	
**Alcoholic status:**	☐ Current consumer<br>☐ Quit alcohol<br>☐ Non-alcoholic (never)

Medical History

**5. TB History:**	
**Have you had TB before?**	☐ Yes ☐ No
**Do you have any history of TB in your family?**	☐ Yes ☐ No<br>If yes, please specify relation: ___________
**Do you have any Known contact with TB patients?**	☐ Yes ☐ No<br>If yes, please provide details: ___________

Symptom Onset and Duration:

**Date of onset of TB symptoms:**		1.___________<br>(dd/mm/yy)<br>2. I don't know/remember<br>___________ days/weeks
**Date of initial medical consultation:**		___________ (dd/mm/yy)
**Duration between onset of TB symptoms and first medical consultation:**		___________ days/weeks
**What symptoms are you experienced prior to diagnosis?**		
**Types of symptoms experienced**	**Presence/Absence**	**Duration of symptoms before seeking medical help**
• **Cough**	☐ Yes ☐ No<br>☐ Dry cough ☐ Cough with bloody sputum ☐ Cough with non-bloody sputum	___________ days/weeks
• **Fever**	☐ Yes ☐ No<br>☐ Persistent fever ☐ Evening rise of temperature ☐ Intermittent fever ☐ Intermittent + Evening rise	___________ days/weeks
• **Chest pain**	☐ Yes ☐ No	___________ days/weeks
• **Shortness of breath**	☐ Yes ☐ No	___________ days/weeks
• **Night sweats**	☐ Yes ☐ No	___________ days/weeks
• **Weight loss**	☐ Yes ☐ No	___________ kg / ___________ days/weeks

• **Fatigue**	☐ Yes ☐ No	__________ days/weeks
• **Hemoptysis (Blood in sputum)**	☐ Yes ☐ No	__________ days/weeks
• **Others (specify)**	☐ Yes ☐ No	__________ days/weeks
**6. Co-Morbidities and Risk Factors:**		
	**Presence/Absence**	**Duration**
**HIV infection**	☐ Yes ☐ No<br>☐ Reactive ☐ non-reactive<br>☐ Unknown	
**Diabetes mellitus**	☐ Yes ☐ No	
**Hypertension**	☐ Yes ☐ No	
**Chronic obstructive pulmonary disease (COPD)**	☐ Yes ☐ No	
**Asthma**	☐ Yes ☐ No	
**Chronic kidney disease**	☐ Yes ☐ No	
**Malnutrition**	☐ Yes ☐ No	
**Cancer (e.g., lung cancer)**	☐ Yes ☐ No	
**Others (specify)**	☐ Yes ☐ No	

Health seeking behaviour among Tuberculosis patients Healthcare Visits and Interventions:

**Healthcare facilities visited before diagnosis:**	
**First action to illness**	☐ Visit Health-Care Facility<br>☐ Self-medication<br>☐ Consult Health Worker<br>☐ Pharmacy / Chemist<br>☐ Use Traditional Medicine<br>☐ Other ___________

**Type of Facility First Visited**	☐ Tuberculosis Treatment Center<br>☐ Health Center (Clinic)<br>☐ Public Hospital<br>☐ Private Hospital<br>☐ Other ___________
**Distance of nearest public health facility from home (in km):**	☐ <1 km<br>☐ 1–3 km<br>☐ >3 km
**Total number of healthcare facilities visited before diagnosis:**	☐ 1 ☐ 2–3 ☐ >3
**Number of visits to HCF before TB diagnosis:**	☐ 1 visit<br>☐ 2–3 visits<br>☐ >3 visits
**Mode of transport used to reach healthcare facility:**	☐ Walk<br>☐ Public transport (Bus, Auto-rickshaw, Shared taxi)<br>☐ Private transport (Bicycle, Motorcycle/Scooter, Car)<br>☐ Other ___________
**Treatments provided (if antibiotics were prescribed before TB diagnosis):**<br><br>**(Note: This information must be extracted from health records — not from patient interviews or memory cues.)**	☐ Yes ☐ No<br><br>If yes, specify (from health records):<br>• Name of antibiotic(s): ______________________<br>• Duration prescribed: ___________ days/weeks

Factors affecting diagnostic delay or Reasons for any delays in diagnosis or treatment

Category	Factors
	**Diagnostic delay**
**Patient-Related Factors**	☐ Poverty and low income<br>☐ Unemployment<br>☐ Catastrophic costs<br>☐ Low awareness of TB symptoms<br>☐ Stigma and fear of isolation<br>☐ Crowded living conditions<br>☐ Gender disparities in accessing care<br>☐ Cultural beliefs and practices<br>☐ Busy occupation / work-related barriers<br>☐ Transportation difficulties / long distance<br>☐ Migration / seasonal mobility<br>☐ Delay in seeking care from a higher-level provider after leaving a previous provider<br>☐ Other (specify) ___________

**Provider-related factors**	☐ Low clinical suspicion of tuberculosis by healthcare providers<br>☐ Prescribing unnecessary antibiotics before TB tests<br>☐ Misdiagnosis as another condition<br>☐ Delayed referrals by healthcare providers<br>☐ Other (specify) ___________
**Treatment delay**	
**Patient-Related Factors**	☐ Poor knowledge of TB treatment protocols<br>☐ Long distance from health facility<br>☐ Fear of long TB treatment duration<br>☐ Reluctance to begin TB treatment due to prolonged duration<br>☐ Other (specify) ___________
**Provider-related factors**	☐ Loss to follow-up after TB diagnosis<br>☐ Delayed initiation after diagnosis due to administrative or logistical issues<br>☐ Other (specify) ___________

KNOWLEDGE AND PERCEIVED STIGMA RELATED TO TUBERCULOSIS

S.N	Questions	Options/Response
**TB Knowledge**		
1.	**Have you previously heard about Tuberculosis (TB)?**	☐ Yes ☐ No
2.	**In your opinion, how serious a disease is TB? (Check one.)**	☐ Very serious<br>☐ Somewhat serious<br>☐ Not very serious
3.	**How serious a problem do you think TB is in your country/region? (Check one.)**	☐ Very serious<br>☐ Somewhat serious<br>☐ Not very serious
4.	**Is TB hereditary?**	☐ Yes<br>☐ No<br>☐ Do not know
5.	**Which organ of the body is most commonly affected by TB?”**	☐ Lungs<br>☐ Heart<br>☐ Liver<br>☐ Kidneys<br>☐ Do not know
6.	**What are the signs and symptoms of TB?**	☐ Persistent cough (≥2 weeks)<br>☐ Fever<br>☐ Chest pain<br>☐ Weight loss<br>☐ Night sweats<br>☐ Fatigue<br>☐ Hemoptysis (coughing blood)<br>☐ Loss of appetite<br>☐ Other — ___________<br>☐ Do not know
7.	**Is TB contagious?**	☐ Yes<br>☐ No<br>☐ Do not know

8.	**How can a person get TB? (Please check all that apply):**	☐ Through handshakes<br>☐ Through the air when a person with TB coughs or sneezes<br>☐ Through sharing dishes<br>☐ Through eating from the same plate<br>☐ Through touching items in public places (doorknobs, handles in transportation, etc.)<br>☐ Do not know<br>☐ Other — ___________
9.	**How can a person prevent getting TB? (Please check all that apply):**	☐ Avoid shaking hands<br>☐ Cover mouth and nose when coughing or sneezing<br>☐ Avoid sharing dishes<br>☐ Wash hands after touching items in public places<br>☐ Keep windows closed at home<br>☐ Maintain good nutrition<br>☐ By praying<br>☐ Do not know<br>☐ Other — ___________
10.	**In your opinion, who can be infected with TB? (Please check all that apply):**	☐ Anybody<br>☐ Only poor people<br>☐ Only homeless people<br>☐ Only alcoholics<br>☐ Only drug users<br>☐ Only people living with HIV/AIDS<br>☐ Only people who have been in prison<br>☐ Other — ___________
11.	**Can TB be cured?**	☐ Yes<br>☐ No<br>☐ Do not know
12.	**How can someone with TB be cured? (Check all that apply):**	☐ Herbal remedies<br>☐ Home rest without medicine<br>☐ Praying<br>☐ Specific drugs given by health center<br>☐ DOTS (Directly Observed Treatment, Short-course)<br>☐ Do not know<br>☐ Other — ___________
13.	**Do you know the approximate duration of TB treatment?**	☐ Yes → If yes, how long?<br>☐ 2 months<br>☐ 6 months<br>☐ 9 months<br>☐ 12 months<br>☐ Other — ___________<br>☐ No
14.	**Do you know the kinds of TB drugs?**	☐ Yes → If yes, specify: ___________<br>☐ No<br>☐ Do not know

15.	**Do you know if there is a vaccine for TB?**	☐ Yes → If yes, name the vaccine: __________<br>☐ No<br>☐ Do not know

Knowledge Scoring and Classification (For Data Collectors / Reviewers Only)

- **Scoring method:**
  - Each correct response = **1 point**
  - Incorrect / wrong / "Do not know" = **0 points**
  - For multiple-response items (e.g., signs & symptoms, prevention, cure), give **1 point per correct option**, up to the predefined maximum. The score for each of these items should be calculated as:
    Respondent's score ÷ Maximum score × 100.

    1 = ≥50% of total score
    0 = <50% of total score
- **Total possible score:** Sum of all items (range: 0–15).

Knowledge classification:

- **Good knowledge = score at or above the median**
- **Poor knowledge = score below the median**

TB Stigma

16.	Do you feel ashamed for having TB?	☐ Agree ☐ Neutral / Not sure ☐ Disagree
17.	Do you have to hide your TB diagnosis from the other people?	☐ Agree ☐ Neutral / Not sure ☐ Disagree
18.	Does TB affect relationships with others?	☐ Agree ☐ Neutral / Not sure ☐ Disagree
19.	Is TB very costly due to the long duration of the disease?	☐ Agree ☐ Neutral / Not sure ☐ Disagree
20.	Do you prefer to live isolated since you got TB diagnosis?	☐ Agree ☐ Neutral / Not sure ☐ Disagree
21.	Does the TB affect your work performance?	☐ Agree ☐ Neutral / Not sure ☐ Disagree
22.	Does TB affect marital relations?	☐ Agree ☐ Neutral / Not sure ☐ Disagree
23.	Does TB affect family responsibilities?	☐ Agree ☐ Neutral / Not sure ☐ Disagree
24.	Do you think there are fewer chances of marriage due to a TB diagnosis?	☐ Agree ☐ Neutral / Not sure ☐ Disagree
25.	Does TB affect your family relations?	☐ Agree ☐ Neutral / Not sure ☐ Disagree
26.	Does TB cause female infertility?	☐ Agree ☐ Neutral / Not sure ☐ Disagree
27.	Does TB lead to serious complications during pregnancy?	☐ Agree ☐ Neutral / Not sure ☐ Disagree
28.	Does TB affect breastfeeding?	☐ Agree ☐ Neutral / Not sure ☐ Disagree
29.	Does TB affect pregnancy outcomes?	☐ Agree ☐ Neutral / Not sure ☐ Disagree
30.	Is a girl unable to decide for getting TB treatment?	☐ Agree ☐ Neutral / Not sure ☐ Disagree

Stigma Scoring and Classification (For Data Collectors / Reviewers Only)

- **Scoring method:**
  - Responses are scored as:
    - **Agree** = 0
    - **Neutral / Not sure** = 0
    - **Disagree** = 1
  - Each "Disagree" reflects **absence of stigma** (positive attitude).
- **Total possible score:** Sum of all items (range: 0–15).

Stigma classification:

- **Low stigma = score at or above the median**
- **High stigma = score below the median**

Diagnosis and Treatment Timeline:

**Date of confirmed TB diagnosis:**	Date: __________ (dd/mm/yy)
**Type of TB:**	☐ Pulmonary TB<br>☐ Extra-pulmonary TB<br>☐ Both
**Place of TB diagnosis:**	☐ Public health facility<br>☐ Private health facility
**Sputum Smear Microscopy [Base-line]:**	
**Findings (AFB):**	☐ Present ☐ Absent
**Report (AFB grading per high-power field):**	☐ Scanty ☐ 1+ ☐ 2+ ☐ 3+
**Gene Xpert (CBNAAT) results:**	
**Date of test:**	__________ (dd/mm/yy)
**MTB detected:**	☐ Yes ☐ No
**Bacterial load:**	☐ Very Low ☐ Low ☐ Medium ☐ High
**Rifampicin resistance detected:**	☐ Yes ☐ No ☐ Indeterminate
**Date of treatment initiation:**	Date: __________ (dd/mm/yy)
**Duration between diagnosis (CBNAAT confirmed) and treatment initiation:**	__________ days/weeks

Diagnostic Facility [Final TB Diagnosis]

**Location:**	**PKTB Sanatorium**
Distance to the facility from patient's residence	
Modes of transportation available and used	
Travel time to reach the facility	
