# Supplementary material for: Patient and health system related factors affecting diagnostic and treatment delays in tuberculosis: A cross-sectional study in Mysuru, South India
Source: PLoS One. 2026 Feb 12;21(2):e0342998. doi: 10.1371/journal.pone.0342998 (PMC12900347; doi:10.1371/journal.pone.0342998)
Supplement: Attachment — Submitted filename: comments from reviewer.docx [file pone.0342998.s002.docx]

**Reviewer’ comment**

| **S.N** | **Heading/subheading of the section** | **Statement by author** | Remarks |
| --- | --- | --- | --- |
| 1 | Abstract/ **Methods** | A community-based cross-sectional retrospective study was conducted at the Princess  Krishnajammanni Tuberculosis and Chest Diseases (PKTB) Sanatorium | clarification is required as the study was done at at the PKTB) Sanatorium, not in the community,  hence study design is centre based not the community based |
| 2 | Abstract/Result | system delay was linked to daily wage occupation (AOR: 6.15; 95% CI: 1.81–20.85), | What were the attributes or factors considered under health system delay , as occupation and below poverty-line status, marital status etc cannot be considered as a risk factor for health system delay as it is more related to delays at patient level. |
| 3 | Abstract /conclusion | Nearly two-thirds of TB patients experienced diagnostic or treatment delay | Need to explain whether 2/3^rd^ of TB patients experienced diagnostic or treatment delay, as both are different things, factors responsible for delay and putting on treatment are different |
| 4 | MATERIALS AND METHODS | Study designed mentioned is retrospective and  The study population comprised newly diagnosed TB patients aged ≥18 years who gave written informed consent and were available for interviews during the study period | Why author has mentioned retrospective study if new cases were enrolled.. **selection criteria needs** clarification in terms of inclusion/ exclusion criteria and sampling technique used, as the centre is offering IPD/OPD based services  From where the cases were enrolled. |
| 5 | MATERIALS AND METHODS /Sample size and selection | prevalence of approximately 40.5%,  a margin of error of 5% | This formula fits more for community based study not the centre based one, current study is centre based. This margin error was relative precision or absolute precision, needs clarification. |
| 6 | **Results** | Sociodemographic characteristics of the study participants | Table 1 can be removed to avoid duplication |
| 7 | **Results** | Clinical characteristics/  Factors associated with patient/diagnostic/treatment/health system delay | **(1)Needs to figure out of total 331 interviewed cases, what were presenting symptoms and the risk factors for delays ( patients/health system) in context for** microbiologically confirmed cases of pulmonary and extra pulmonary Tb cases, and factors should analysed separately, for all types of delays patient/diagnostic/treatment /health system delays for pulmonary and extra pulmonary Tb cases. |
| 8 | **Results** | Table 1 to 5 | Table 1 to 5 can be omitted to avoid duplication as summarized through text only, table number 8 where Factors associated with health system delay among tuberculosis patient has been depicted.  Table/s can be placed at the end after the references . |
| 9 | **Discussion** |  | it is lengthy, needs to make concise and crispy |
| 10 | Strengths and limitations of the study | It includes a large, diverse sample that strengthens the reliability and generalizability of findings within the regional context | This is single centre based Study with sample size of 331 so results can be generalized so lacks external validity. |

Following are the comments

1. In the Abstract/ **Methods: author has mentioned** A community-based cross-sectional retrospective study was conducted at the PrincessKrishnajammanni Tuberculosis and Chest Diseases (PKTB) Sanatorium

**Comment**

Clarification is required as the study was done at at the PKTB) Sanatorium, not in the community, hence study design is centre based not the community based.

2. Abstract/Result author has mentioned system delay was linked to daily wage occupation (AOR: 6.15; 95% CI: 1.81–20.85

Comment: What were the attributes or factors considered under health system delay , as occupation and below poverty-line status, marital status etc cannot be considered as a risk factor for health system delay as it is more related to delays at patient level.

3. Abstract /conclusion, author has mentioned Nearly two-thirds of TB patients experienced diagnostic or treatment delay

Comment: Need to explain whether 2/3^rd^ of TB patients experienced diagnostic or treatment delay, as both are different things, factors responsible for delay and putting on treatment are different.

4. MATERIALS AND METHODS , author has mentioned, Study designed mentioned is retrospective and the study population comprised newly diagnosed TB patients aged ≥18 years who gave written informed consent and were available for interviews during the study period.

Comment: Why author has mentioned retrospective study if new cases were enrolled.. **selection criteria needs** clarification in terms of inclusion/ exclusion criteria and sampling technique used, as the centre is offering IPD/OPD based services From where the cases were enrolled.

5. MATERIALS AND METHODS /Sample size and selection, author has mentioned prevalence of approximately 40.5%,a margin of error of 5%

Comment: This formula fits more for community based study not the centre based one, current study is centre based. This margin error was relative precision or absolute precision, needs clarification.

6. **Results, author has given description for** Sociodemographic characteristics of the study participants.

**Comment:** Table 1 can be removed to avoid duplication

Clinical characteristics/

7. In the result , Factors associated with patient/diagnostic/treatment/health system delay has been reflected.

Comment : **1)Needs to figure out of total 331 interviewed cases, what were presenting symptoms and the risk factors for delays ( patients/health system) in context for** microbiologically confirmed cases of pulmonary and extra pulmonary Tb cases, and factors should analysed separately, for all types of delays patient/diagnostic/treatment /health system delays for pulmonary and extra pulmonary Tb cases

8. Comment: Table 1 to 5 can be omitted to avoid duplication as summarized through text only, table number 8 where Factors associated with health system delay among tuberculosis patient has been depicted.
9. Table/s can be placed at the end after the references .
10. Discussion is i is lengthy, needs to make concise and crispy
11. Strengths and limitations of the study : It includes a large, diverse sample that strengthens the reliability and generalizability of findings within the regional context.

Comment : This is single centre based Study with sample size of 331 so results can be generalized so lacks external validity.
