# Supplementary material for: Patient and health system related factors affecting diagnostic and treatment delays in tuberculosis: A cross-sectional study in Mysuru, South India
Source: PLoS One. 2026 Feb 12;21(2):e0342998. doi: 10.1371/journal.pone.0342998 (PMC12900347; doi:10.1371/journal.pone.0342998)
Supplement: Attachment — Submitted filename: Response to Reviewers.docx [file pone.0342998.s003.docx]

**Factors affecting diagnostic and treatment delays in tuberculosis: A cross-sectional study in Mysuru, south India**

**Response to Reviewers**

**Reviewer #1:**

**Methodology**
**Comment:** The manuscript presents a contradiction by describing the study as "community-based" in the abstract and methods section, while simultaneously acknowledging it as "single-center, facility-based" in the limitations. Recruitment exclusively from a tertiary-care hospital inherently defines the study as facility-based, which impacts the generalizability of the results. This terminology should be clarified for consistency to accurately reflect the source of participants and the implications for external validity.

**Response:** We thank the reviewer for pointing out the inconsistency in terminology. We acknowledge that the use of “community-based” was incorrect, as all participants were recruited exclusively from PKTB Hospital. We have now corrected the study classification throughout the manuscript to “hospital-based cross-sectional retrospective study.” Additionally, we have clarified that PKTB is a specialized secondary-care tuberculosis and chest disease facility. These revisions ensure accurate representation of the study setting and improve clarity regarding the generalizability of the findings.

**Comment:** While "Diagnostic delay" and "Treatment delay" are adequately defined, the relationship between these and the "Patient delay" and "Health system delay" components, which sum to "Total delay," needs explicit clarification in the text. Relying solely on Figure 2 for this crucial relationship could lead to misinterpretation. The definitions should clearly state how "Diagnostic delay" and "Treatment delay" are situated within or relate to "Patient delay" and "Health system delay," respectively.

**Response:** We agree that these temporal components must be explicitly and textually defined rather than depending solely on Figure 2. We have now revised the definitions in the Methods section to clearly articulate how “Patient delay” and “Health system delay” contribute to “Total delay,” and how “Diagnostic delay” and “Treatment delay” are respective components within these intervals. These clarifications provide clearer conceptual relationships and prevent misinterpretation.

**Results and Statistical Analysis**

**Comment:** The multivariable logistic regression models frequently yield extremely large Adjusted Odds Ratios (AORs) accompanied by exceptionally wide 95% Confidence Intervals (CIs). For instance, an AOR of 31.40 (95% CI: 3.46–284.74) for poor TB knowledge or 24.81 (95% CI: 10.34–59.50) for visiting more than three facilities indicates significant model instability, likely due to small cell sizes (quasi-complete separation). Presenting such point estimates without acknowledging this high imprecision is misleading. The discussion notably omits the interpretation of this instability. Future analyses may consider more robust statistical methods, such as Firth regression or Exact Logistic Regression to produce more stable and reliable estimates.

**Response:** We thank the reviewer for this important statistical observation. We agree that the large AORs and wide confidence intervals reflect imprecision due to sparse data in certain subgroups and potential quasi-complete separation. In response, we have revised the manuscript to explicitly acknowledge this limitation in both the Results and Discussion sections. Additionally, we have added a statement suggesting that future analyses with larger sample sizes or the application of penalized (Firth) or Exact Logistic Regression may improve estimate stability. While we did not re-estimate models using these methods in the current manuscript, we have clarified the interpretive caution necessary for interpreting large AORs with wide CIs.

**Comment:** The practice of dichotomising continuous variables, such as knowledge and stigma scores, at their median is statistically suboptimal. This approach results in the loss of valuable data. Analysing these variables as continuous predictors (with appropriate checks or transformations) or utilising multiple ordered categories (e.g., quartiles) would provide a more complete and statistically sound understanding of their relationships with the outcomes.

**Response:** We thank the reviewer for this methodological insight. We agree that dichotomising continuous variables (such as knowledge and stigma scores) at the median leads to information loss and may reduce statistical sensitivity. We have now acknowledged this limitation in the manuscript and clarified that the dichotomisation was chosen to facilitate interpretability and consistency with similar TB delay studies. We also note that future analyses with larger datasets will consider modelling these variables in their continuous form or using multiple categories (e.g., tertiles/quartiles) to enhance statistical precision. And most importantly at this stage, we have not re-coded the analysis using continuous variables, as this would alter the statistical structure of the current findings. However, we fully recognise the impact of dichotomisation and have incorporated this acknowledgment into the limitations. The point raised will directly inform our future analytical design for subsequent datasets.

**Comment:** The use of a p-value-based stepwise selection approach (p≤0.05 in bivariate analysis for inclusion in multivariable models) can lead to model instability, omitted-variable bias, and potentially spurious findings. A more robust and recommended approach involves selecting variables based on theoretical relevance or employing a less stringent univariate screening threshold (e.g., p≤0.20) to ensure the inclusion of important confounders in the multivariable models.

**Response:** We thank the reviewer for highlighting this important statistical consideration. We acknowledge that using a p≤0.05 threshold for variable inclusion may be restrictive and could contribute to model instability and omitted-variable bias. We have now acknowledged this limitation in the manuscript and clarified that our selection strategy followed a conservative threshold approach, but future analyses will consider variable selection based on theoretical relevance and/or a broader inclusion criterion (such as p≤0.20) to ensure more comprehensive adjustment for confounding.

**Discussion**
**Comment:** The discussion notes that factors like age, poverty, occupation, stigma, and smoking, initially associated with patient delay, lost significance after multivariable adjustment, suggesting indirect effects. To strengthen this observation, the discussion should elaborate on how these factors might operate indirectly or identify the most likely mediators in the study's specific context. For example, exploring whether they primarily influence informal education or TB-related knowledge could provide deeper contextual insights.

**Response:** We thank the reviewer for this insightful suggestion. In response, we have expanded the discussion to explore potential indirect pathways by which variables such as age, socioeconomic status, occupation, smoking, and stigma may influence patient delay indirectly through mediators like TB-related knowledge, health-seeking behaviour, informal education, or social support. These additions strengthen contextual interpretation and provide plausible mechanistic explanations within the study setting.

**Comment:** The discussion presents an intriguing paradox: while initially seeking care at private facilities is associated with significantly higher odds of delay (AOR: 17.28), being diagnosed at a private facility appears to be protective against delay in treatment initiation (AOR: 0.072). The discussion should provide a more explicit and detailed explanation of this distinction. It needs to clarify whether this implies that initial private consultations often involve misdiagnosis or a lack of TB services, thus causing delays. However, once a diagnosis is established within a private setting, the administrative process for treatment initiation is faster.

**Response:** We thank the reviewer for identifying this important and apparently paradoxical finding. We have now expanded the discussion to explain this distinction more clearly. We propose that initial consultations at private clinics or pharmacies may contribute to patient delay or diagnostic delay due to low suspicion of TB, nonspecific symptom treatment, or lack of diagnostic infrastructure. In contrast, once a formal TB diagnosis is established in a private facility, treatment initiation may proceed rapidly due to fewer administrative steps and more direct referral pathways. These clarifications have been incorporated into the discussion to contextualize the observed contrasting effects.

**Reviewer #2:**

**Comment:** Clarification is required as the study was done at at the PKTB) Sanatorium, not in the community, hence study design is center based not the community based.

**Response:** We thank the reviewer for this correction. We agree that the study was conducted at PKTB Hospital and is appropriately classified as a hospital/center-based study. We have revised the terminology accordingly in the Abstract, Methods, and elsewhere in the manuscript to ensure consistency and accuracy. The term “community-based” has been removed and replaced with “hospital-based and or facility-based,” and we have clarified that all participants were recruited from PKTB, a specialty center for tuberculosis and chest diseases.

**Comment**: What were the attributes or factors considered under health system delay, as occupation and below poverty-line status, marital status etc cannot be considered as a risk factor for health system delay as it is more related to delays at patient level.

**Response:** We thank the reviewer for this important observation. We agree that variables such as occupation, marital status, and poverty-line classification represent patient-level socioeconomic characteristics rather than health system determinants. To clarify this conceptual distinction, we have added a statement in the Methods section indicating that these variables were included as patient-level predictors that may indirectly influence the likelihood of experiencing health system delay—for example, through patterns of healthcare utilization or repeated facility visits—and not as constitutive components of health system delay itself.

**Comment:** Need to explain whether 2/3rd of TB patients experienced diagnostic or treatment delay, as both are different things, factors responsible for delay and putting on treatment are different.

**Response:** We thank the reviewer for raising this point. We agree that diagnostic delay and treatment delay are distinct concepts with different determinants. In our data, the proportion approaching two-thirds refers specifically to *diagnostic delay* and not a combined measure of diagnostic and treatment delays. We have revised the Abstract conclusion accordingly to state “Nearly two-thirds experienced diagnostic delay,” thereby removing ambiguity and accurately reflecting the observed pattern.

**Comment:** Why author has mentioned retrospective study if new cases were enrolled. selection criteria need clarification in terms of inclusion/ exclusion criteria and sampling technique used, as the Centre is offering IPD/OPD based services from where the cases were enrolled.

**Response:**
Thank you for this important observation. Although newly diagnosed TB patients were enrolled during the study period, the study is classified as retrospective because information on delays (symptom onset, health-seeking behaviour, and prior diagnostic pathways) was collected based on past events recalled by patients, rather than prospectively observed. We have also stated that participants were recruited consecutively from both OPD and IPD services. Inclusion and exclusion criteria were already provided in the “Study Participants” section; however, we have strengthened this description to avoid ambiguity.

**Comment:** This formula fits more for community based study not the Centre based one, current study is Centre based. This margin error was relative precision or absolute precision, needs clarification.

**Response:** Thank you for highlighting this methodological concern. The sample size formula applied was based on the estimated prevalence of diagnostic delay reported in prior TB studies (≈40.5%), with a 5% absolute precision. We acknowledge that this approach is commonly used in community-based prevalence studies, but we applied it here to estimate the required number of newly diagnosed TB patients within the facility during the study period. To improve clarity, we have now specified that the 5% margin of error refers to absolute precision, and we have revised the Methods section accordingly to avoid misinterpretation.

**Comment:** Table 1 can be removed to avoid duplication

**Response:** We appreciate the reviewer’s suggestion. However, Table 1 provides essential sociodemographic variables that are not fully presented in the narrative text, including occupation categories, education levels, socioeconomic classification, and household background characteristics. Removing the table would result in the loss of these critical descriptive details. Additionally, presenting sociodemographic characteristics in tabular form is consistent with standard epidemiological reporting formats and aligns with practices observed in similar TB delay publications. Therefore, we respectfully request to retain Table 1 for clarity, completeness, and ease of interpretation.

**Comment :** 1)Needs to figure out of total 331 interviewed cases, what were presenting symptoms and the risk factors for delays ( patients/health system) in context for microbiologically confirmed cases of pulmonary and extra pulmonary Tb cases, and factors should analyzed separately, for all types of delays patient/diagnostic/treatment /health system delays for pulmonary and extra pulmonary Tb cases

**Response:**
We thank the reviewer for this insightful comment. All presenting clinical symptoms for both pulmonary and extrapulmonary TB cases are already comprehensively presented in Table 2. As noted, a substantial proportion of extrapulmonary TB patients also exhibited classical respiratory symptoms typically associated with pulmonary TB (e.g., cough, breathlessness). Because of this symptom overlap, classification based on presenting symptoms alone would introduce misclassification bias. Therefore, we categorized patients anatomically as pulmonary vs. extrapulmonary TB based on standardized microbiological diagnostic criteria rather than symptom profiles.

Furthermore, microbiological confirmation and sample size were predominantly available for pulmonary TB, while the number of extrapulmonary cases was limited (n=14). Thus, performing stratified regression analyses of risk factors across four types of delays for EPTB would not be statistically robust and may create unstable estimates. However, if the editor and reviewers consider it essential, we can add an additional supplementary table differentiating the symptom distribution and delay-related characteristics between PTB and EPTB for descriptive purposes.

**Comment:** Table 1 to 5 can be omitted to avoid duplication as summarized through text only, table number 8 where Factors associated with health system delay among tuberculosis patient has been depicted.

**Response:** We thank the reviewer for this suggestion. While we appreciate the concern regarding potential redundancy, Tables 1–5 present detailed categorical distributions (including sociodemographic, clinical, knowledge, stigma, and healthcare-seeking variables) that are only partially summarized in narrative form. Many of these data points cannot be fully conveyed through text alone without loss of detail. Additionally, the presentation of descriptive characteristics in tabular format is consistent with standard epidemiological reporting and based on this rationale and referencing structural formatting used in comparable published studies, we respectfully request to retain Tables 1–5 to ensure clarity, transparency, and completeness of the dataset.

**Comment:** Table/s can be placed at the end after the references.

**Response:** We thank the reviewer for the suggestion. We understand the request to place all tables at the end of the manuscript. However, we respectfully request to retain the tables within the Results section. Presenting results and corresponding tables together allows the reader to directly correlate the narrative findings with the supporting data, minimizing the need to repeatedly scroll back and forth to the end of the document. We believe this improves clarity, reduces potential confusion, and maintains logical flow.

Additionally, we have reviewed several recently published articles in PLOS ONE within the tuberculosis and infectious disease domain, and many of these retain tables within the Results section to aid readability and continuity of interpretation. We are, of course, willing to adjust the formatting if it necessary, but we believe the current placement supports clearer communication of the findings.

**Comment:** Discussion is i is lengthy, needs to make concise and crispy

**Response:** We thank the reviewer for this helpful suggestion. We agree that a concise discussion improves clarity. Accordingly, we carefully revised and trimmed the Discussion to make it more succinct, removed repetitive explanatory text, and focused on essential interpretative elements of the findings.

We also wish to note that some of our co-authors had initially reviewed the manuscript and requested more detailed elaboration in certain parts of the Discussion to ensure contextual clarity for readers, and one of the reviewers also suggested the need for elaboration on certain interpretations. Therefore, the Discussion originally contained additional explanation to maintain scientific transparency and reproducibility of interpretation. Nonetheless, we have now struck a balance between brevity and clarity, ensuring the Discussion remains informative without being unnecessarily long.

**Comment:** This is single center-based Study with sample size of 331 so results can be generalized so lacks external validity.

**Response:** We thank the reviewer for the important feedback. We have now revised the Strengths and Limitations section accordingly. Specifically, we have removed any implication of external generalizability and revised the text to state that the study includes a diverse patient sample that enhances the reliability and internal consistency of the findings within our regional setting. Additionally, we have clearly acknowledged that, due to the single-center design, the external validity of the results is limited. These revisions ensure accurate interpretation and maintain appropriate scientific rigor.
