# Supplementary material for: Patient and health system related factors affecting diagnostic and treatment delays in tuberculosis: A cross-sectional study in Mysuru, South India
Source: PLoS One. 2026 Feb 12;21(2):e0342998. doi: 10.1371/journal.pone.0342998 (PMC12900347; doi:10.1371/journal.pone.0342998)
Supplement: Attachment — Submitted filename: comments from reviewer_PLOS ONE.docx [file pone.0342998.s004.docx]

**Reviewer’ comment**

| **Heading/subheading of the section** | **Statement by author** | Earlier Remarks | ss  Current status |
| --- | --- | --- | --- |
| Abstract/ **Methods** | A community-based cross-sectional retrospective study was conducted at the Princess  Krishnajammanni Tuberculosis and Chest Diseases (PKTB) Sanatorium | clarification is required as the study was done at at the PKTB) Sanatorium, not in the community,  hence study design is centre based not the community based | Corrections done |
| Abstract/Result | system delay was linked to daily wage occupation (AOR: 6.15; 95% CI: 1.81–20.85), | What were the attributes or factors considered under health system delay , as occupation and below poverty-line status, marital status etc cannot be considered as a risk factor for health system delay as it is more related to delays at patient level. | Not corrected in abstract,  While occupation & marital related factors were considered as patient related factor in material & methods ( study variables) |
| Abstract /conclusion | Nearly two-thirds of TB patients experienced diagnostic or treatment delay | Need to explain whether 2/3^rd^ of TB patients experienced diagnostic or treatment delay, as both are different things, factors responsible for delay and putting on treatment are different | Corrected |
| MATERIALS AND METHODS | Study designed mentioned is retrospective and  The study population comprised newly diagnosed TB patients aged ≥18 years who gave written informed consent and were available for interviews during the study period | Why author has mentioned retrospective study if new cases were enrolled. **selection criteria needs** clarification in terms of inclusion/ exclusion criteria and sampling technique used, as the centre is offering IPD/OPD based services  From where the cases were enrolled. | Not corrected, retrospective design is still there, still it is not clear out of 331 cases enrolled, how many were taken from OPD and IPD in revised MS. |
| MATERIALS AND METHODS /Sample size and selection | prevalence of approximately 40.5%,  a margin of error of 5% | This formula fits more for community based study not the centre based one, current study is centre based. This margin error was relative precision or absolute precision, needs clarification. | As study is already done so can be ignored now. |
| **Results** | Sociodemographic characteristics of the study participants | Table 1 can be removed to avoid duplication |  |
| **Results** | Clinical characteristics/  Factors associated with patient/diagnostic/treatment/health system delay | **(1)Needs to figure out of total 331 interviewed cases, what were presenting symptoms and the risk factors for delays ( patients/health system) in context for** microbiologically confirmed cases of pulmonary and extra pulmonary Tb cases, and factors should analysed separately, for all types of delays patient/diagnostic/treatment /health system delays for pulmonary and extra pulmonary Tb cases. |  |
| **Results** | Table 1 to 5 | Table 1 to 5 can be omitted to avoid duplication as summarized through text only, table number 8 where Factors associated with health system delay among tuberculosis patient has been depicted.  Table/s can be placed at the end after the references . | Table 1-5 Not removed,  As these information is repetition in subsequent tables so table 6,7, 8 can be retained |
| **Discussion** |  | it is lengthy, needs to make concise and crispy | Revised and corrected |
| Strengths and limitations of the study | It includes a large, diverse sample that strengthens the reliability and generalizability of findings within the regional context | This is single centre based Study with sample size of 331 so results can be generalized so lacks external validity. | Revised and corrected |

Following are the revised comments for Factors Affecting Diagnostic and Treatment Delays in Tuberculosis: A Cross-Sectional Study in Mysuru, South India

1. Title reflects health system delays however patient related delay is missing. for health system delays which mainly includes diagnosis and treatment delays or slowing down timely diagnosis and treatment after a patient seeks care. Which is within healthcare infrastructure, inadequate resources, provider practices, and patient flow, including limited diagnostic tools (like X-ray, smear tests), poor referral systems between public/private sectors, insufficient clinician awareness of diseases (like TB), geographical barriers, long wait times, author has mentioned some of them correctly that Health system delay was linked to first seeking care at private facilities (AOR: 17.28; 95% CI: 5.86–50.94), and visiting more than three healthcare facilities before diagnosis, ) **however as per earlier comment that Health system delay was linked to daily wage occupation (AOR: 6.15; 95% CI: 1.81–20.85), below-poverty-line economic status (AOR: 13.89; 95% CI: 3.38–57.17) is still leading to confusion to consider it as patient related delay rather than Health system delay.**
2. **In results about Sociodemographic characteristics of the study participants have been already mentioned as per earlier comments to avoid repetition table 1 can be removed.**
3. **Regarding Clinical characteristics in result section as per earlier remark author has mentioned only symptoms related to pulmonary TB, however there were 14 Extra pulmonary TB cases it would be interesting to know what was the presenting or clinical finding.**

Overall comment : Maximum comments have been addressed and by incorporating the minor suggestions study can be accepted for publication.
