# Supplementary material for: Patient and health system related factors affecting diagnostic and treatment delays in tuberculosis: A cross-sectional study in Mysuru, South India
Source: PLoS One. 2026 Feb 12;21(2):e0342998. doi: 10.1371/journal.pone.0342998 (PMC12900347; doi:10.1371/journal.pone.0342998)
Supplement: Attachment — Submitted filename: Response_to_Reviewers_auresp_2.docx [file pone.0342998.s005.docx]

**Factors affecting diagnostic and treatment delays in tuberculosis: A cross-sectional study in Mysuru, south India**

**Response to Reviewers**

**Reviewer #1:**

**Comment:** The manuscript presents a timely and important study on the factors affecting diagnostic and treatment delays in tuberculosis in Mysuru, South India. I recommend the publication of this manuscript.

**Response:** We sincerely thank the reviewer for the positive feedback and for recommending our manuscript for publication. We greatly appreciate the time and effort taken to review our work.

**Reviewer #2:**

**Comment:** Title reflects health system delays however patient related delay is missing. for health system delays which mainly includes diagnosis and treatment delays or slowing down timely diagnosis and treatment after a patient seeks care. Which is within healthcare infrastructure, inadequate resources, provider practices, and patient flow, including limited diagnostic tools (like X-ray, smear tests), poor referral systems between public/private sectors, insufficient clinician awareness of diseases (like TB), geographical barriers, long wait times, author has mentioned some of them correctly that Health system delay was linked to first seeking care at private facilities (AOR: 17.28; 95% CI: 5.86–50.94), and visiting more than three healthcare facilities before diagnosis, ) however as per earlier comment that Health system delay was linked to daily wage occupation (AOR: 6.15; 95% CI: 1.81–20.85), below-poverty-line economic status (AOR: 13.89; 95% CI: 3.38–57.17) is still leading to confusion to consider it as patient related delay rather than Health system delay.

**Response:** We thank the reviewer for this important and thoughtful comment. We agree that health system delay is conventionally defined as delays arising from healthcare infrastructure, provider practices, diagnostic availability, referral pathways, and system-level processes after a patient first seeks care.

In our analysis, socioeconomic variables such as daily wage occupation and below-poverty-line (BPL) status were not treated as components of the health system itself, but rather as patient-level characteristics that influence interactions with the health system after initial care-seeking. These factors were included as explanatory variables in the health system delay model because they affect patients’ ability to navigate healthcare services, attend repeated visits, comply with referrals, and access diagnostics within the health system.

To address this conceptual concern and avoid misclassification, we have clarified this distinction explicitly in the *Study Variables* and *Discussion* sections. We now emphasize that daily wage work and poverty function as patient-level determinants of health system delay, rather than intrinsic health system failures. Additionally, we have revised the title and relevant text to better reflect the dual contribution of patient-related and health system–related delays. These revisions improve conceptual clarity and align our findings with established frameworks for tuberculosis care delays.

**Comment**:  In results about Sociodemographic characteristics of the study participants have been already mentioned as per earlier comments to avoid repetition table 1 can be removed.

**Response:** We thank the reviewer for this constructive suggestion and agree that unnecessary repetition should be avoided. However, after careful consideration, we have retained Table 1, as it provides a comprehensive and structured presentation of baseline sociodemographic and lifestyle characteristics that are essential for contextualizing the study population and interpreting the regression analyses. Importantly, Table 1 includes key variables—such as occupation categories, education levels, socioeconomic classification, and household background characteristics—that are not fully detailed in the narrative text, and removing the table would result in the loss of critical descriptive information. To address concerns regarding redundancy, we have streamlined the Results section to include only a brief summary of key characteristics, with detailed distributions presented exclusively in Table 1. In addition, during internal review, several coauthors independently recommended retaining Table 1, as it enhances clarity, completeness, and ease of interpretation for readers. Therefore, we respectfully request the reviewer’s approval to retain Table 1 in the revised manuscript.

**Comment:** Regarding Clinical characteristics in result section as per earlier remark author has mentioned only symptoms related to pulmonary TB, however there were 14 Extra pulmonary TB cases it would be interesting to know what was the presenting or clinical finding.

**Response:** We thank the reviewer for this valuable suggestion. We agree that inclusion of clinical features among extrapulmonary tuberculosis (EPTB) cases would provide a more comprehensive description of the study population. Accordingly, we have added a brief description of the presenting clinical features of extrapulmonary TB cases in the Results section under *Clinical characteristics*. This addition improves completeness.
