# Supplementary material for: Patient and health system related factors affecting diagnostic and treatment delays in tuberculosis: A cross-sectional study in Mysuru, South India
Source: PLoS One. 2026 Feb 12;21(2):e0342998. doi: 10.1371/journal.pone.0342998 (PMC12900347; doi:10.1371/journal.pone.0342998)
Supplement: Attachment — Submitted filename: Third time reviewed and Comments from Dr Rashmi Sharma.docx [file pone.0342998.s006.docx]

| S.N | Last comments | Corrections in revised MS |
| --- | --- | --- |
| 1 | Cross-Sectional Study in Mysuru, South India 1. Title reflects health system delays however patient related delay is missing. for health system delays which mainly includes diagnosis and treatment delays or slowing down timely diagnosis and treatment after a patient seeks care. Which is within healthcare infrastructure, inadequate resources, provider practices, and patient flow, including limited diagnostic tools (like X-ray, smear tests), poor referral systems between public/private sectors, insufficient clinician awareness of diseases (like TB), geographical barriers, long wait times, author has mentioned some of them correctly that Health system delay was linked to first seeking care at private facilities (AOR: 17.28; 95% CI: 5.86–50.94), and visiting more than three healthcare facilities before diagnosis, ) however as per earlier comment that Health system delay was linked to daily wage occupation (AOR: 6.15; 95% CI: 1.81–20.85), below-poverty-line economic status (AOR: 13.89; 95% CI: 3.38–57.17) is still leading to confusion to consider it as patient related delay rather than Health system delay. | Corrected and now the revised title is Patient and health system related factors affecting diagnostic and treatment delays in tuberculosis. |
| 2 | In results about Sociodemographic characteristics of the study participants have been already mentioned as per earlier comments to avoid repetition table 1 can be removed. | Corrections not done , still it is existing |
| 3 | Regarding Clinical characteristics in result section as per earlier remark author has mentioned only symptoms related to pulmonary TB, however there were 14 Extra pulmonary TB cases it would be interesting to know what was the presenting or clinical finding. | Corrections done and symptoms of Extrapulmonary Tb have been mentioned |
